# Recurrent Falls Due to Hypoglycemia: Case of an IGF-2-producing Fibrous Tumor of the Pleura

**DOI:** 10.1210/jcemcr/luae061

**Published:** 2024-04-25

**Authors:** Mathijs Cornelis Guijt, David J Heineman, Jacqueline Thérèse Jonker

**Affiliations:** Department of Internal Medicine, Haga Ziekenhuis Den Haag, Els Borst-Eilersplein 275, 2545 AA, Den Haag, The Netherlands; Department of Surgery and Cardiothoracic Surgery, Amsterdam UMC location Vrije Universiteit Amsterdam—Cancer Center Amsterdam, De Boelelaan 1117, 1081 HV, Amsterdam, The Netherlands; Department of Internal Medicine, Alrijne Ziekenhuis Leiderdorp, Simon Smitweg 1, 2353 GA, Leiderdorp, The Netherlands

**Keywords:** hypoglycemia, IGF-2, big IGF-2, NICTH

## Abstract

This case report delineates the clinical presentation of a 77-year-old male who experienced falls and sustained a humerus fracture attributed to hypoglycemia. Despite the absence of insulin use and normal laboratory results for cortisol, TSH, blood count, and liver and kidney function, a fasting test revealed diminished C-peptide and insulin levels, ruling out insulinoma, exogenous insulin use, or β-cell hyperplasia. Subsequent laboratory investigations demonstrated lowered IGF-1 and elevated IGF-2 levels, indicative of an IGF-2-producing tumor as the etiology of the hypoglycemia. A positron emission tomography computed tomography scan identified a right-sided thoracic cavity tumor, prompting an open resection. Postoperatively, hypoglycemic episodes abated within 2 days, and pathology confirmed a 14.9-cm solitary fibrous tumor. Nonislet cell tumor hypoglycemia (NICTH), also known as Doege Potter syndrome, arises from aberrant production of IGF-2 or its precursors. Elevated IGF-2 levels induce hypoglycemia through heightened glucose uptake on binding to insulin receptors. The literature supports the efficacy of both surgical intervention and corticosteroids in managing NICTH. This case underscores the importance of considering NICTH as a rare etiology in unexplained hypoglycemia cases, advocating for the utility of fasting tests in diagnosis, and suggesting surgical resection as a viable treatment option when radical excision is feasible.

## Introduction

Nonislet cell tumor hypoglycemia (NICTH) is an uncommon syndrome characterized by recurrent hypoglycemic episodes resulting from the ectopic production of IGF-2 (1). In contrast to hypoglycemia associated with insulinoma, NICTH does not involve islet cell hyperplasia or excessive insulin secretion. Rather, it emanates from nonpancreatic tumors producing IGF-2, which binds to insulin receptors, provoking heightened insulin-like activity and subsequent hypoglycemia (1). Although exceedingly uncommon, the precise prevalence of NICTH remains uncertain (1).

IGF-2, a pivotal growth factor regulating fetal and postnatal growth, bears structural similarities to insulin (2). In the context of NICTH, tumors such as mesenchymal tumors, adrenal tumors, and hepatocellular carcinomas exhibit overexpression of IGF-2, disrupting glucose metabolism.

Diagnosing NICTH poses challenges because of its infrequency and nonspecific symptoms. Patients typically manifest hypoglycemia-related symptoms, including confusion, diaphoresis, and seizures. Pinpointing the root cause necessitates a comprehensive diagnostic approach, involving assessments of insulin, C-peptide, IGF-1, and IGF-2 levels, complemented by imaging modalities to identify the tumor responsible for IGF-2 production.

This case report provides an overview of a patient with NICTH attributed to an IGF-2-producing tumor of the visceral pleura, shedding light on the diagnostic complexities, management approaches, and long-term outcomes. Through heightened awareness and comprehension of this rare condition, we aspire to facilitate early recognition, accurate diagnosis, and effective management of NICTH.

## Case Presentation

This case report details the presentation of a 77-year-old male who initially sought medical attention because of falls, resulting in a proximal humerus fracture. He had experienced dizziness for several weeks. After surgical intervention, he was discharged and subsequently admitted to a nursing home for rehabilitation. During an outpatient clinic visit with his internist about 3 weeks later, the physician observed that the patient had experienced hypoglycemia on the night of sustaining the fracture.

While at the rehabilitation center, a recurring tendency to fall was noted, prompting glucose monitoring on request. Severe nocturnal hypoglycemia was identified, necessitating the patient to wake up nightly to consume a snack as a preventive measure. Apart from the hypoglycemic episodes and a predisposition to falls, the patient reported no other complaints, and his weight remained stable. Nutritional and family history was unremarkable. His life style had not changed over the past few years. His medical history included hypertension, hypercholesterolemia, a transient ischemic attack, and benign prostate hypertrophy. Notably, the patient did not use insulin, sulfonylurea, or glinide medications, but was on a regimen of amlodipine, lisinopril, simvastatin, tamsulosin, and clopidogrel.

## Diagnostic Assessment

On physical examination, the patient had some verrucae on the skin without clinical significance on consultation of a dermatologist. The remainder of the physical examination was unremarkable. Laboratory examination found mild normocytic anemia with normal count of thrombocytes and leukocytes (hemoglobin: 8.3 mmol/L [13.4 g/dL]) (normal range, 8.5-11.0 mmol/L); mean corpuscular volume: 90 fL (90 µm³) (normal range, 80-100 fL); thrombocytes: 174 × 10⁹/L (174 × 10³/mm³) (normal range 150-400 × 10⁹/L); leukocytes: 8.3 × 10⁹/L (8.3 × 10³/mm³) (normal range, 4.0-10.0 × 10⁹/L); normal renal function (creatinine: 71 µmol/L [0.8 mg/dL]) (normal range, 64-104 µmol/L); estimated glomerular filtration rate (CKD-EPI formula: 86 mL/min/1.73 m²) with normal sodium, potassium, and mild hyperchloremia (sodium: 144 mmol/L [144 mEq/L]) (normal range, 135-145 mmol/L); potassium: 3.6 mmol/L (3.6 mEq/L) (normal range, 3.5-5.1 mmol/L); and chloride: 116 mmol/L (116 mEq/L) (normal range, 96-107 mmol/L). TSH and morning cortisol levels were normal (cortisol: 0.222 µmol/L [8.05 µg/dL]) (normal range, 0.150-0.555 µmol/L) and TSH: 1.96 mU/L (1.96 μIU/mL) (normal range, 0.27-4.20 mU/L). Although alkaline phosphatase and lactate dehydrogenase were slightly elevated, other liver markers were normal (bilirubin total: 15 µmol/L [0.88 mg/dL]) (normal range, 3-20); alkaline phosphatase: 244 U/L (4.07 µkat/L) (normal range, 0-115); gamma glutamyl transpeptidase: 22 U/L (0.37 µkat/L) (normal range, 0-55); aspartate transaminase: 17 U/L (0.28 µkat/L) (normal range, 0-35); alanine aminotransferase: 8 U/L (0.13 µkat/L) (normal range, 0-45); lactate dehydrogenase: 271 U/L (4.5 µkat/L) (normal range, 0-248); albumin: 35.7 g/L (3.57 g/dL) (normal range, 35-55); total protein: 63.1 g/L (6.31 g/dL) (normal range, 60.0-80.0); and C-reactive protein: 1 mg/L (100 µg/dL) (normal range, 0-5).

Based on the history, physical examination, and laboratory investigations, potential causes such as alcohol abstinence; starvation; insulin or oral antidiabetic drug abuse; hepatic, renal, or cardiac failure; sepsis; and hypocortisolism were deemed unlikely contributors to the hypoglycemia in this specific case. It is noteworthy that, in general, medication errors are more probable culprits for hypoglycemia than is an IGF-2-producing tumor. A test for the use of secretagogue medications or insulin was conducted and yielded negative results.

To further distinguish between an insulin-dependent or insulin-independent form of endogenous hypoglycemia in this patient, a fasting test was administered. Remarkably, after just 2 hours of fasting, a glucose level of 2.3 mmol/L (41.4 mg/dL) (normal range, 3.5-7.8 mmol/L) was measured, accompanied by suppressed C-peptide and insulin levels (C-peptide: 0.05 nmol/L [0.15 ng/mL]) (normal range, 0.26-0.62 nmol/L) and insulin: <2.0 mmol/L (<0.28 µIU/mL) (normal range, 0-29.1 mmol/L). This fasting test result diminished the likelihood of an insulinoma, β-cell hyperplasia (nesidioblastosis) or autoimmune-mediated cause as an explanation for the hypoglycemia.

**Figure 1.**
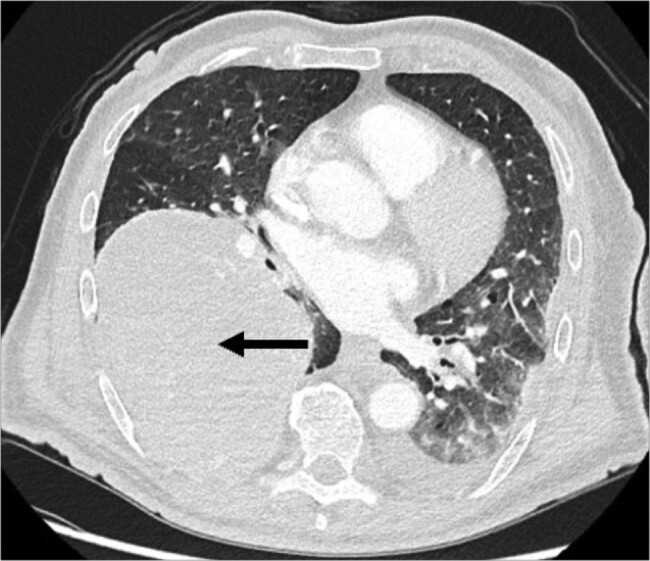
Computed tomography scan. A large solid mass of the right pleura with a diameter of 14 × 14 × 11 cm located above the diaphragm.

Having ruled out other potential causes, IGF-1/IGF-2 determination and a positron emission tomography computed tomography (PET-CT) was performed. The PET-CT scan revealed a substantial solid mass on the right pleura, measuring 14 × 14 × 11 cm in diameter, situated above the diaphragm. Notably, the lesion exhibited limited fluorodeoxyglucose (FDG) avidity, primarily evident laterally at the periphery (Fig. 1). Radiologically, this observation aligns with characteristics indicative of a solitary fibrous tumor. Additionally, enlarged lymph nodes paratracheal and subcarinal were identified, which did not display FDG avidity.

## Treatment

Because of the unmanageable nature of hypoglycemia symptoms in the nursing home, the patient was transferred to the hospital. On admission, a continuous 10% glucose infusion was initiated to control hypoglycemia. Following the PET-CT results, the patient was referred to a university hospital with a pulmonary surgery unit, with the provisional diagnosis of a solitary IGF-2-producing tumor originating from the right basal pleura. An open resection of the pleural tumor was subsequently performed.

Preoperatively, uncertainty existed regarding whether the tumor infiltrated the lung parenchyma or originated from the parietal pleura; therefore, a low posterolateral thoracotomy was carried out in the sixth intercostal space. During surgery, it was observed that the tumor originated from the visceral pleura of the right lower lobe, and the lobe was not attached to the thoracic wall or the diaphragm. Consequently, an open lobectomy of the right lower lobe was undertaken, accompanied by a systematic lymph node dissection. After resection, a section of the seventh rib was excised to facilitate the extraction of the substantial specimen.

The postoperative recovery period transpired without complications. Nocturnal hypoglycemia showed improvement, allowing the discontinuation of glucose infusion and prednisone, which had been initiated because of an escalation in nocturnal hypoglycemia before surgery. The recovery process involved the participation of physiotherapy and a dietitian to enhance the patient's postsurgery condition.

## Outcome and Follow-up

Within 2 days after surgery, the hypoglycemia abated, and the patient no longer exhibited any related symptoms. Slightly over a week later, the patient was discharged from the hospital, and within a few weeks, a complete recovery was achieved. Roughly 1 month following the surgical intervention, the results of the IGF-1 and IGF-2 determinations affirmed our initial diagnosis of an IGF-2-producing tumor, revealing suppressed levels of IGF-1 and elevated levels of IGF-2 (IGF-1: 7.2 nmol/L [55.1 ng/mL]; IGF-2: 90.3 nmol/L [689 ng/mL]).

**Figure 2.**
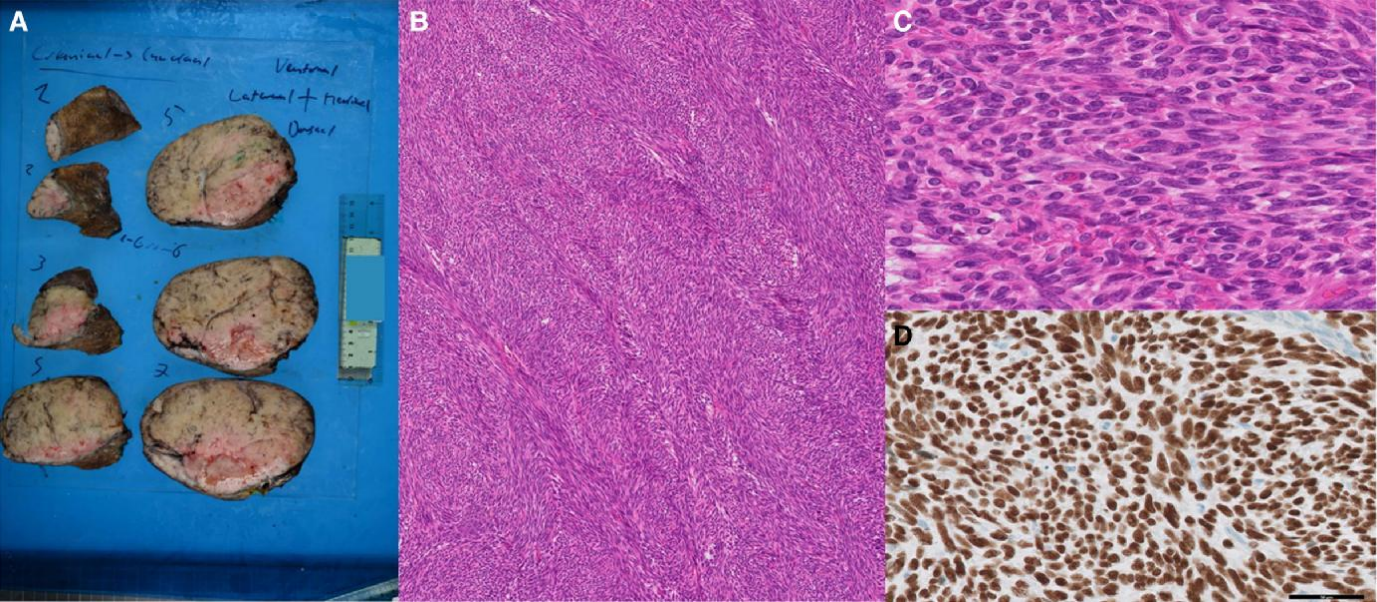
Histology of solitary fibrous tumor. Macroscopy showed a large solid mass of the right pleura with a diameter of 14 × 14 × 11 cm (A). Histology showed a fascicular proliferation of monomorphic, bland spindle cells without prominent mitotic activity and no necrosis (B, C). Tumor cells showed strong nuclear positivity for STAT6 immunohistochemistry confirming the diagnosis of solitary fibrous tumor (D).

The pathology report documented the successful resection of a solitary fibrous tumor measuring 14.9 cm in diameter, with no indications of malignant transformation (refer to Fig. 2 for macro- and microscopic images). Subsequent IGF-1 and IGF-2 determinations performed several months after surgery indicated the normalization of IGF-1 and IGF-2 levels (IGF-1: 21.3 nmol/L [162.9 ng/mL]; IGF-2: 50.8 nmol/L [388 ng/mL]).

The patient was discharged from the nursing home and resumed living in his own house, a significant milestone for his overall well-being. During a follow-up outpatient clinic visit with his internist a few months later, the patient reported being able to ride his bike again and noted the absence of the need for a midnight snack since the surgery.

## Discussion

In this case, the hypoglycemia was treated, and the IGF-1/IGF-2 ratio was normalized by resecting a large solitary fibrous tumor in the right lower lobe. The ectopic production of IGF-2 from this tumor is known as the Doege-Potter syndrome, named after the 2 authors who described it in 1930. A literature review on this syndrome revealed that these tumors are often not FDG avid, as in our case, because of elevated levels of IGF-2 in the muscle, liver, and heart (3). Most of these tumors originate from the pleural cavity, followed by the pelvis. In this literature review, 61% of the cases were malignant.

Most studies on NICTH identify surgery as the most effective treatment for hypoglycemia. A retrospective cohort study by Rena et al included 21 patients, 14.3% of whom had NICTH. In all these patients, hypoglycemic symptoms disappeared after surgery, and IGF-1 and IGF-2 ratios normalized if known (4). The tumors described in this study measured from 22 × 12 × 8 to 330 × 280 × 190 mm (4). Overall, according to our case, we can conclude that these tumors tend to be large in size.

A case series by Taele et al described 8 patients with NICTH and various underlying tumors (5). Five patients received prednisone, whereas 3 patients underwent surgery, 1 of whom had prior prednisone. Prednisone was successful in 5 of the 6 patients, and the dosage was patient-specific, ranging between 5 and 60 mg per day. Again, these case series showed that hypoglycemia ceased in all operated patients, corresponding with our patient.

In the latest case series of 6 patients with NICTH by Jannin et al, 3 patients underwent successful surgery without a return of hypoglycemia (6). In 1 patient, the hypoglycemia had initially disappeared but later recurred because of an IGF-2-producing recurrence, requiring reoperation. These 3 patients all had a solitary fibrous tumor of the pleura. Prednisone was effective in bridging the gap until surgery. In 3 patients with a different tumor type, prednisone was effective in controlling hypoglycemia only in a neuroendocrine tumor of the palate, not in the other 2 (retroperitoneal myxofibrosarcoma and meningeal hemangiopericytoma). This shows that the effectiveness of prednisone in the treatment of NICTH is likely tumor dependent. A limitation for both our case report and the case series by Jannin et al is that the pattern of the dose-dependent effect of prednisone remains unclear. Investigating this would be an important topic for further studies. For the treatment of malignant solitary fibrous tumors that cannot be radically resected, adjunctive therapies including immunotherapy and antiangiogenics can be considered depending on tumor characteristics (7).

This case report illustrates the diagnostic pathway, treatment, and outcome of a patient with NICTH caused by an IGF-2-producing tumor. Despite its rarity, this diagnosis belongs in the differential diagnosis of hypoglycemia. For clinicians, it is useful to realize that a fasting test can be used to properly differentiate between different causes of hypoglycemia. Clinicians should be aware that they should always exclude medication errors or abuse by testing for the use of secretagogue medications or insulin. The cornerstone of the treatment of NICTH is tumor surgery. If this is not possible or to prevent hypoglycemia as a bridge to surgery, prednisone can be considered in a dosage that depends on patient and tumor factors that are difficult to predict.

## Learning Points

- Despite its rarity, clinicians should consider the diagnosis of nonislet cell tumor hypoglycemia (NICTH) caused by an IGF-2 producing tumor as part of the differential diagnosis of hypoglycemia.
- Clinicians should be aware that they must exclude medication errors or abuse by testing for the use of secretagogue medications or insulin.
- A fasting test can assist clinicians in differentiating between various causes of hypoglycemia.
- The cornerstone of NICTH treatment is tumor surgery.
- If surgery is not possible or as a bridge to surgery, glucocorticoids can be considered.
- Future studies should focus on understanding the challenging dose-dependent effects of glucocorticoids in NICTH to enhance the treatment of unresectable patients.

## Data Availability

Original data generated and analyzed for this case report are included in this published article.

## Abbreviations

FDG: fluorodeoxyglucose
NICTH: nonislet cell tumor hypoglycemia
PET-CT: positron emission tomography computed tomography
